# GridScore: a tool for accurate, cross-platform phenotypic data collection and visualization

**DOI:** 10.1186/s12859-022-04755-2

**Published:** 2022-06-06

**Authors:** Sebastian Raubach, Miriam Schreiber, Paul D. Shaw

**Affiliations:** 1 Department of Information and Computational Sciences, The James Hutton Institute, Invergowrie, Dundee, Scotland; 2 Department of Life Science, University of Dundee, Dundee, Scotland

**Keywords:** Phenotyping, Data-visualization, Data-collection, Data-transfer, Bioinformatics, Plant-genetics, Plant-breeding

## Abstract

**Background:**

Plant breeding and crop research rely on experimental phenotyping trials. These trials generate data for large numbers of traits and plant varieties that needs to be captured efficiently and accurately to support further research and downstream analysis. Traditionally scored by hand, phenotypic data is nowadays collected using spreadsheets or specialized apps. While many solutions exist, which increase efficiency and reduce errors, none offer the same familiarity as printed field plans which have been used for decades and offer an intuitive overview over the trial setup, previously recorded data and plots still requiring scoring.

**Results:**

We introduce GridScore which utilizes cutting-edge web technologies to reproduce the familiarity of printed field plans while enhancing the phenotypic data collection process by adding advanced features like georeferencing, image tagging and speech recognition. GridScore is a cross-platform open-source plant phenotyping app that combines barcode-based systems with a guided data collection approach while offering a top-down view onto the data collected in a field layout. GridScore is compared to existing tools across a wide spectrum of criteria including support for barcodes, multiple platforms, and visualizations.

**Conclusion:**

Compared to its competition, GridScore shows strong performance across the board offering a complete manual phenotyping experience.

## Background

Efficient and accurate phenotypic data collection underpins large areas of plant breeding and crop research [1]. It is therefore essential that mechanisms that facilitate and drive the data collection process are in place to ensure that the foundation upon which plant research is based is as stable and accurate as possible. Historically, the recording of plant phenotyping data has evolved from basic hand-written records on printed field plans through rudimentary data collection applications or spreadsheets run on portable devices, to advanced, feature-rich, and specialized applications running on mobile phones and tablets. Each approach exhibits both unique advantages and potential sources of errors. Hand-written notes are quick and familiar but require an additional digitization step and are prone to errors such as typos, number swaps and field layout mix-ups. Illegible or simply unfamiliar handwriting further impede the digitization step. Spreadsheets are the natural next step forward from hand-written notes and are a widely used tool for data recording. They offer basic mechanisms for data verification with most users familiar with their operation. When used on mobile devices outdoors, spreadsheets have major limitations relating to the accurate input of data due to the application complexity, sunlight, and often awkward input methods. In addition, more advanced features which are important like image tagging or geolocation are not available.

Modern mobile applications (apps) represent a current approach towards efficient manual plant phenotyping. They aim to work on low-cost mobile devices and address many of the issues that previous approaches exhibit. While tools such as Germinate Scan [2] try to reduce user input to a minimum by completely adopting barcodes for the identification of plants as well as traits and phenotypes, apps like FieldBook [3], KDSmart [4], PhenoApp [5] and Phenobook [6] use a guided approach taking the user through the field plot by plot in a defined order. What these apps lack is the intuitiveness of hand-written notes and printed field plans where data was recorded in each cell of the plan and related easily to the field layout. This approach allowed immediate identification of plots requiring scoring and how trait data compares between plots as well as facilitating easier navigation through the field layout. Outside of the field of phenotyping, data collection frameworks like the OpenDataKit (ODK) [7] have gained popularity. While ODK provides an excellent infrastructure for data collection in the field, it is too abstract to be directly useful for phenotyping. The lack of specificity directly contradicts the need for a fast system closely tailored to the specific requirements. A logical next step is an application that would harmonize all three approaches—bar coding, guided data recording and a field-layout-centric overview—into a single tool that is fast, easy to set up and intuitive to use. To this end, we propose a new phenotyping app which we call ‘GridScore’.

## Implementation

GridScore is a cross-platform open-source plant phenotyping app combining a visual view of the field layout with barcode system compatibility and a guided data collection approach allowing step-by-step collection of phenotypic data. GridScore is a Progressive Web App (PWA) written in Vue.js [8] and supported by a Java [9] and MySQL [10] server-side to facilitate configuration and data sharing across devices. GridScore uses cutting-edge web technology standards such as reactive interfaces, usage of the Web Speech API (Application Programming Interface), support for High Dots Per Inch (HiDPI) displays and IndexedDB [11] for the local storage of data on the device.

## Results and discussion

### Installation, setup and usage

GridScore runs on any modern phone, PC or tablet across all major platforms. Installing GridScore is achieved by either bookmarking the website or using the browser’s install feature to pin it to the user’s home screen from which point onwards it acts in the same way as other native applications. GridScore is also available using a native Android wrapper and can conveniently be installed through the Google Play Store. All data generated and stored using GridScore is held locally on the device ensuring its offline availability and removing the need for a mobile data signal or wireless network connectivity while collecting data. When connectivity is available, data can be synchronized between devices using the GridScore server architecture allowing data to be collected on multiple devices. This is achieved by communicating with the central GridScore server or, optionally, a local instance of GridScore using the official Docker [12] container.

**Fig. 1:**
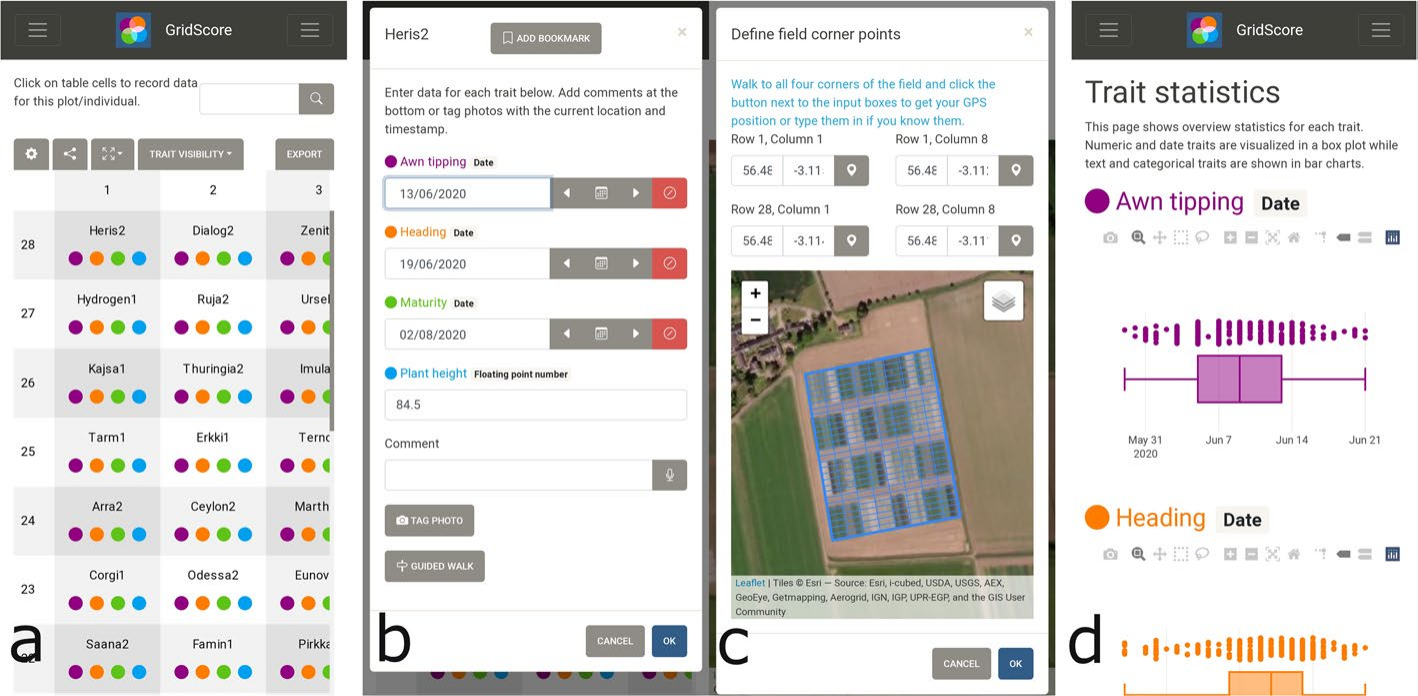
The GridScore user interface. **a** The main data input screen of GridScore reflects a field plan with rows and columns. Each cell represents a plot/pot, and the colored dots indicate which traits have already been scored. **b** Selection of a plot opens the data input dialog. Data is entered for each trait using a data-type-specific input field. Image tagging, bookmarking and guided walks are available from this screen. **c** Defining the GPS coordinates of the field corner points results in a field plan overview overlaid onto a map. **d** Data visualizations including statistics are available at any time during data collection. All charts can be downloaded for presentations and publications

The process of using GridScore can be split into four parts: setup, data collection, data visualization and data sharing. The setup step requires, at a minimum, a distinctive trial name, the number of rows and columns in the field layout, the list of planted germplasm/replicates and the list and data types of traits scored during the trial. GridScore uses a row by column field layout as this was shown through user shadowing to be the most natural and commonly used trial layout, however, alternative layouts can be accommodated. The list of germplasm is used to fill the layout with unique germplasm identifiers. If available, these can be linked to barcodes located in the trial to simplify the plot identification by scanning the appropriate barcode, which reduces the error rate for misidentification of plots. Traits can be of the following types: floating point number, integer, categorical, date or free text. GridScore will adapt the data input mechanism to the data type of each trait accordingly by showing a numeric input keyboard, drop-down options or calendar inputs. As a data validation mechanism, range restrictions can be assigned to numeric traits and valid categories for categorical traits must be pre-defined. Data type enforcement alongside restrictions allow for early error prevention during the data collection process. Invalid data input will be highlighted and rejected. Repeated measurements of the same trait for a single plot can be enabled by selecting the multi-measurement trait type. Optionally, to support geographic position referencing within trials, the corner points of the field layout can be defined either manually, through selection on a map, or by visiting the site. These trial boundaries together with information on numbers of rows and columns are fed into a perspective projection [13] onto the surface of the earth (Fig. 1c). The projection allows the pinpointing and highlighting of the current GPS (Global Positioning System) location, giving an accurate position of where data is collected as well as providing the user with a reference point within the field plan showing their current position and aiding in the navigation of the trial.

To simplify the setup process, trials can be configured on a PC using GridScore in a web browser and can then be shared to mobile devices by generating and scanning a QR (Quick Response) code. This code uniquely identifies the trial setup and allows facile sharing of identical configurations to other devices. The main data entry interface of GridScore consists of a tabular representation of the field layout where each cell represents a plot or a pot (Fig. 1a). Recorded data is indicated in each cell by a uniquely colored circle (using color blind safe palettes) for each trait. This allows quick identification of the data collection progress per trait as well as plots that still require data collection.

### Data collection and visualization

Data collection is achieved by identifying the current plot either by its row and column number, via the GPS position indicator, by scanning a barcode identifying the plot or by using the guided data collection mode. Offering these four options presents flexibility with regards to the collection of data using personal preference. Selecting the plot opens the data entry dialog (Fig. 1b) which, for each trait, shows appropriate input fields based on the data types. Dates can be chosen from a calendar or stepped through via dedicated buttons. Categorical traits are either selectable from a drop-down box or, for a category with few values, selectable via individual toggle buttons. Data input is audibly repeated via text-to-speech technology giving immediate feedback and allowing manual verification and correction of data. Predefined valid ranges and category values are checked on each data input and errors are clearly highlighted. Each data point is associated with the timestamp at recording as well as the user’s exact geographic location. In addition to predefined traits, free text comments can be added to data points. GridScore offers this feature via direct text input or speech recognition, where a user’s speech is converted into text and stored alongside the recorded data. Photographs are often used to record characters of interest, like unusual phenotypes or weather events, as well as to generate a catalog of individuals during a trial. If taken directly with a digital camera or a phone, these photographs need to be renamed or mapped back to the individual within the trial in a post-processing step, requiring additional time and effort and potentially introducing preventable errors. GridScore can be used to take photographs of plants during the data collection step which are automatically renamed with the germplasm identifier and timestamp as well as tagged with the GPS location. If photographs are taken with dedicated digital cameras, GridScore can associate them back to the data at a later stage. In addition to the manual identification of the current plot, GridScore offers a ‘guided walk’ option where one of eight navigational sequences through the field is chosen from any starting position and the user is consequently guided through the field along this route.

As well as the data recording aspect, GridScore includes data visualizations offering overviews of the scoring progress and distribution of trait values. A line chart shows the percentage of plots scored per trait over time which acts both as a progress measure, and an indicator for temporal relationships between traits. Additionally, trait values can be visualized using a heat map which can highlight possible environmental impacts on the trial such as soil properties or edge effects. Another view shows statistical values per trait in the form of box plots for numeric and date traits, and bar charts for categorical traits emphasizing possible outliers and clusters (Fig. 1d). Optionally, bar charts along the side of the field plan can be enabled to highlight the scoring progress per column and row for each trait.

### Data export and sharing

Data is exported in a text-based, tab-delimited format. Each row contains the trait data for a specific germplasm/replicate along with GPS coordinates and timestamp. Trait definitions can be exported together with their data ranges and categories. The formats used are compatible with (but not restricted to) the plant genetic resources database Germinate [14] and allow easy import into Excel or any processing pipeline. Using the same QR code process used to share trial setups, the newly scored data can be shared back to the user’s PC in the same way to avoid having to manually handle files. Breeding API (BrAPI) [15] compatibility currently exists for trait import and we are planning to expand on this in the future to include data export to any BrAPI-compatible database.

### Exemplar uses of GridScore

GridScore has been evaluated across species including barley, potato and blackcurrants and in different environments (e.g., fields, glasshouses and poly-tunnels). The generic and adaptable design of GridScore has allowed it to be applied effectively to these scenarios. In blackcurrants, traits like yield are not scored once, but repeatedly across the season, which is achieved by regularly exporting and resetting the trial. In barley, GridScore has been used on multiple projects to score traits ranging from growth habit to plant height—measured using a barcoded measuring stick - to date traits such as emergence, heading date and maturity. To support the Commonwealth Potato Collection (CPC) [16], GridScore was used to extend the image catalog by automatically tagging images with the appropriate germplasm and timestamp.

### Comparison to other tools

The area of app-assisted phenotypic data collection has seen a significant interest in recent years and other applications have been developed to assist in the data collection process. Approaches for data collection can be split into three categories: guided, unstructured and field-plan centric. While guided tools - like KDSmart and FieldBook—aim to steer users through the field one plot at a time in a strict predefined pattern, unstructured tools free themselves from a field layout to offer more flexibility, but consequently lose the very narrow focus of phenotypic data collection. Field-plan-centric approaches assume the viewpoint of a traditional top-down field plan offering the required context for phenotypic data collection while not being too restrictive in their navigation. GridScore is the first app to coin the field-plan-centric approach exploiting the users’ familiarity of field plans while also offering guided walks and unstructured input and therefore supporting all three approaches to offer the most flexibility.

Table 1 shows an overview of representative applications of each type against a list of features. Where required, limitations are added. KDSmart lacks in terms of open-source, cross-platform support and BrAPI support. FieldBook lacks cross-platform support, data visualizations, and the field plan view lacks usability. PhenoApp is not directly available on an app store or online, lacks cross-platform and BrAPI support, visualizations, a field plan view, GPS referencing, voice feedback and requires locally importing Excel template files. Germinate Scan lacks cross-platform support, BrAPI support, visualizations, and a field plan view. Open Data Kit requires complex setup before scoring can start, is not a stand-alone tool, lacks a field plan view, georeferencing, visualizations and BrAPI support. GridScore shows great performance across the board. The only restriction is the currently limited BrAPI support. In all other categories, GridScore scores better or equal to the other tools and significantly advances the capabilities of this class of data collection tools.

**Table 1:** Detailed comparison of data collection applications. Each column represents a phenotypic collection application, and the rows list desirable criteria. Each cell indicates whether the tool fulfils that criterium and to what extent. Plus-signs indicate the full support of a feature while minus-signs mark unavailable features

	GridScore	KDSmart	FieldBook	PhenoApp	Germinate scan	Open data kit
Approach	Field-plan-centric, guided, unstructured	Guided	Guided	Guided	Unstructured	Unstructured
Barcode support	+, camera and scanner	+, camera	+, camera	-	+, camera and scanner	+, camera
Cross-platform	+	–	–	–	–	+
Open-source	+	–	+	+	+	+
Trial generation on device possible	+	+	+	–	+	–
Stand-alone	+	+	+	+	+	–
Field plan view	+	+	–	–	–	–
Progress indicator	+, per column, row, cell and overall	+, per cell	–	–	–	+
Guided mode	+	+	+	+	–	–
Georeferencing (user position)	+, on map and field plan	+, on field plan	–	–	+, on map	–
Visualizations	+, heat map, timeline, trait statistics, scatter plots	+, heat map	–	–	–	–
Voice feedback	+	–	–	–	+	–
BrAPI support	+, limited	–	+	–	–	–

## Conclusions

GridScore is a new plant phenotyping app utilizing state-of-the-art technologies to improve plant phenotypic data collection. By combining the best features of existing approaches and including a new set of advanced functionalities, GridScore delivers a complete package suitable for manual plant phenotyping experiments. We introduced a new category of manual phenotypic data collection tools inspired by printed field plan scoring sheets and enriched by a set of innovative features. Together with support for guided and unstructured data input, GridScore covers all bases and offers users the most flexibility. Compared to a representative selection of existing phenotyping apps, GridScore outperforms the competition across the board.

## Availability and requirements

Project name: GridScore Project home page: https://ics.hutton.ac.uk/gridscore/

Operating system(s): Platform independent

Programming language: JavaScript, Java, SQL

Other requirements: Web browser

License: Apache License, Version 2.0

Any restrictions to use by non-academics: None

## Data Availability

GridScore is open source, freely available and distributed as a Progressive Web App (PWA) across all platforms [17], an Android wrapper is available on Google Play [18]. The source code is available on GitHub [19] and a Docker container is available on Docker Hub [20].

## Abbreviations

API: Application Programming Interface
BrAPI: Breeding API
GPS: Global Positioning System
HiDPI: High Dots Per Inch
ODK: Open Data Kit
PWA: Progressive Web App
QR Code: Quick Response code
